# Up-regulation of GSTT1 in serous ovarian cancer associated with resistance to TAXOL / carboplatin

**DOI:** 10.1186/s13048-021-00873-2

**Published:** 2021-09-17

**Authors:** Jing Zhang, Suhong Xie, Lei Zhou, Xiaoyu Tang, Xiaolin Guan, Minjie Deng, Hui Zheng, Yanchun Wang, Renquan Lu, Lin Guo

**Affiliations:** 1 Department of Clinical Laboratory, Fudan University Shanghai Cancer Center, No.270, Dong’An Road, Xuhui District, Shanghai, 200032 China; 2 Department of Oncology, Shanghai Medical College, Fudan University, Shanghai, China

**Keywords:** *GSTT1*, *Serous* ovarian cancer, *Drug resistance*, *Topo I*

## Abstract

Serous ovarian cancer (SOC) is the most common women cancer and the leading cause of cancer-related mortality among the gynaecological malignancies. Although effective chemotherapeutics combined with surgery are developed for the treatment, the five-year survival rate is unsatisfactory due to chemoresistance. To overcome this shortcoming of chemotherapy, we established taxol and carboplatin resistant SOC cell lines for the understandings of the molecular and cellular mechanisms of chemoresistance. Here, we found that these chemoresistant cell lines showed less viability and proliferation, due to more cells arrested at G0/G1 phase. Glutathione-S-transferases-theta1 (GSTT1) was significantly upregulated in these chemoresistant cells, along with other chemoresistant genes. Meanwhile, GSTT1 expression was also significantly upregulated in the SOC patient tissues after taxol treatment, indicating this upregulation was physiologically relevant to chemotherapy. Further, suppression of GSTT1 expression by shRNA in SOC cell lines led to more sensitivity to drug treatment, through increasing divided cells and promoting cell death. Moreover, the expression of DNA topoisomerase 1 (Topo I) was in synergy with that of GSTT1 in the chemoresistant cells, and GSTT1 can bind to Topo I in vitro, which suggested GSTT1 could function through DNA repair mechanism during chemoresistance. In summary, our data imply that GSTT1 may be a potential biomarker or indicator of drug resistance in serous ovarian cancer.

## Introduction

Ovarian cancer (OC) is the most lethal gynaecologic cancer, which accounts for 4% of all kinds of women’s cancer. The most malignant subtype of ovarian cancer is high-grade serous ovarian cancer (SOC), causing the most lethality of ovarian cancer cases [1, 2]. So far chemotherapy is one of effective treatments for ovarian cancer. Many chemotherapeutics, such as taxol (TAX), carboplatin (CBP), doxorubicin, cyclophosphamide, etc., are widely used in clinical practice, eliminating cancer cells through inhibiting the proliferation of and promoting the death of cancer cells. For example, taxol can combine with tubulin polymers, and selectively block cell proliferation in the G2/M phase, and thus induce cytotoxicity. Carboplatin can alkylate DNA in cancer cells, resulting in the disruption of DNA structure and the death of cancer cells.

Despite advances in chemotherapy, the overall five-year survival rate for SOC is just roughly 20%, mainly due to chemoresistance, which leads to cancer recurrence and treatment failure for SOC patients [3, 4]. Previous studies indicated that cancer chemoresistance is underlined by multiple mechanisms, including DNA repair, autophagy, drug efflux, metabolism reprogramming, epithelial-mesenchymal transition (EMT), mitochondrial alteration, etc. [5, 6]. Molecules involved in those processes may play roles in regulating chemoresistance, and could provide potential targets for drug treatment or diagnosis, in order to improve survival rates for ovarian cancer patients.

To investigate the molecular and cellular mechanisms of ovarian cancer chemoresistance, taxol and carboplatin, two commonly used effective drugs in clinical practice, were utilized for developing chemoresistant SOC cell lines. In this study, we show here that Glutathione-S-transferases-T1 (GSTT1), a member of metabolic enzymes that catalyse the conjugation of glutathione onto endogenous and xenobiotic reactive intermediates [7, 8], and a previously showed cancer related gene, is related to the chemoresistance of SOC cell lines. The expression level of GSTT1 is not only substantially up-regulated in taxol and carboplatin resistant SOC cell lines, but also physiologically up-regulated in the tissues from SOC patients after chemotherapy. Down-regulation of GSTT1 in SOC cells reduced cell viability and increased the sensitivity to treatments, through promoting cell death and increasing divided cells at G2/M phase. Interestingly, DNA topoisomerase 1 (Topo I), which is synergistically up-regulated in drug-resistant SOC cells, could interact with GSTT1, based on our preliminary efforts to illustrate the molecular mechanism of GSTT1’s role in chemoresistance of SOC cell lines.

## Materials and methods

### Ovarian cancer tissues

The study was approved by the Ethics Committee of Shanghai Cancer Center, Fudan University (Certification no. 050432–4-1212B). Prior written informed consent was obtained from all patients. Tissues were obtained from patients who had undergone an operation at Gynecologic Surgery, Fudan University Shanghai Cancer Center (Shanghai, China) between 2013 and 2015, including 40 SOC tissues (FIGO stage II-IV, median age 55 years) and 20 normal tissues (ovary tissues obtained from 20 patients who underwent ovariohysterectomy for other gynecological cancer, median age 58 years). All specimens were collected and frozen in liquid nitrogen immediately after surgery and then stored at − 80 °C until analysis. The diagnoses of all the patients were confirmed by histopathological examination.

### Cell culture and reagents

Human OC cell lines SKOV3 and HO8910 were purchased from Shanghai Chinese Academy of Sciences and were cultured in RPMI-1640 medium (Gibco) containing 10% foetal bovine serum, 1% HEPES and 1% penicillin-streptomycin (Gibco). Cell culture was performed in an incubator with a CO_2_ concentration of 5% and a temperature of 37 °C.

### Establishment of taxol / carboplatin-resistant OC cell lines (SKOV3-TAX/CBP, HO8910-TAX/CBP)

Taxol (TAX, concentration) and carboplatin (CBP) were obtained from the pharmacy department of Fudan University Shanghai Cancer Center. Taxol/carboplatin-resistant OC cell lines (SKOV3-TAX/CBP, HO8910-TAX/CBP) were established in vitro by the gradually increased concentration method with time-stepwise increment. Firstly, SKOV3 and HO8910 cells were exposed to stepwise escalating concentrations of TAX (2.5 μg/ml, 5 μg/ml, 7.5 μg/ml, 10 μg/ml and 12.5 μg/ml) for 48 h, when they grew to 70–80% confluence, to check the initial concertration. The treated cells were then washed with PBS and cultured in TAX-free RPMI-1640 that was refreshed every day. The dead cells were washed out with PBS, and fresh medium again was added daily. After the cells recovered, the initial concentration was checked as 2.5 μg/ml, and TAX was added to the cells at increased doses. While the final concentration of TAX was five-fold higher than the initial concentration, the SKOV3-TAX (12.5 μg/ml) and HO8910-TAX (12.5 μg/ml) cells were treated with low concentrations of CBP (5 μg/ml, 10 μg/ml, 15 μg/ml, 20 μg/ml and 25 μg/ml). The treatment with CBP was repeated the same as previous steps with TAX treatment, until the initial concentration was checked as 5 μg/ml and the final five-fold concentration was reached to 25 μg/ml. Then, this cyclic treatment with TAX and CBP was performed three times, as TAX and CBP respectively was reached to 10-fold, 15-fold and 20-fold higher than the initial concentration in the end (as TAX-50 μg/ml and CBP-100 μg/ml). The resistant cells were then cultured without TAX and CBP for three passages and frozen in the liquid nitrogen [9].

### Establishment of GSTT1 down-regulated transduced cells

Short hairpin RNA (shRNA) sequences (Table 1) were designed with the RNAi Designer program. The segments of nucleotides were cloned into the pLKO.1-puro vector (Addgene, MA, USA). The envelope vector pMD2.G, packing plasmid PAX2 and recombined plasmids were transfected into HEK293T cells at a ratio of 1:3:4. The virus supernatant was collected after 48 h and transduced into the OC cell lines using polybrene (8 mg/ml, Sigma, USA) for 24 h to generate shGSTT1–1 and shGSTT1–2 transduced cells. Transduced cells were screened by puromycin (1 mg/ml, Sigma-Aldrich, MO, USA) for 5 days [10].

**Table 1:** Sequences of primers and targets

Primers/targets	Sequences
GSTT1 qRT-PCR forward	5′-CCAAGCTGCACGATAGGTCAC-3′
GSTT1 qRT-PCR reverse	5′-GGTATGCTACACACAGCTCCAC-3′
MDR1 qRT-PCR forward	5′-GACATGACCAGGTATGCCTA-3′
MDR1 qRT-PCR reverse	5′-CTTGGAGACATCATCTGTAAGTC-3′
Topo I qRT-PCR forward	5′-AGGTTCCTTCTCCTCCTCCA-3′
Topo I qRT-PCR reverse	5′-GCCGAGCAGTCTCGTATTTC-3′
ERCC1 qRT-PCR forward	5′-CCTTATTCCGATCTACACAGAGC-3′
ERCC1 qRT-PCR reverse	5′-TATTCGGCGTAGGTCTGAGGG-3’
Bcl2 qRT-PCR forward	5′-TTGCCAGCCGGAACCTATG-3’
Bcl2 qRT-PCR reverse	5′-CGAAGGCGACCAGCAATGATA-3’
Cyclin B1 qRT-PCR forward	5′-AATAAGGCGAAGATCAACATGGC-3’
Cyclin B1 qRT-PCR reverse	5′-TTTGTTACCAATGTCCCCAAGAG-3’
Cyclin E1 qRT-PCR forward	5′-GCCAGCCTTGGGACAATAATG-3’
Cyclin E1 qRT-PCR reverse	5′-CTTGCACGTTGAGTTTGGGT-3’
β-actin qRT-PCR forward	5′- AAGGTGACAGCAGTCGGTT-3’
β-actin qRT-PCR reverse	5′- TGTGTGGACTTGGGAGAGG-3’
shGSTT1–1 forward	5′-CCGGCAGCACTTAAGCGATGCCTTTCTCGAGAAAGGCATCGCTTAAGTGCTGTTTTTG-3’
shGSTT1–1 reverse	5′-AATTCAAAAACAGCACTTAAGCGATGCCTTTCTCGAGAAAGGCATCGCTTAAGTGCTG-3’
shGSTT1–2 forward	5′-CCGGGCTTGCTTAAGACTTGCCCAACTCGAGTTGGGCAAGTCTTAAGCAAGCTTTTTG-3’
shGSTT1–2 reverse	5′-AATTCAAAAAGCTTGCTTAAGACTTGCCCAACTCGAGTTGGGCAAGTCTTAAGCAAGC-3’

### Cell cycle assay

Following incubation for 24 h, cells were harvested, washed with ice-cold PBS, and fixed with 70% alcohol overnight at 4 °C. The fixed cells were incubated with 500 μl PI (BD PharmingenTM, USA) for 15 min at room temperature in the dark and were analysed by flow cytometry. Cell distributions were assessed using ModFit LT software (Verity Software House, ME).

### Cell death assay

Cells were harvested, resuspended in 0.5 ml binding buffer, and incubated with Annexin V-fluorescein isothiocyanate/PI dual stain (BD Biosciences, San Jose, CA, USA) for 20 min and determined by flow cytometry. The assays were repeated three times. Cells negative for both PI and Annexin V were considered viable cells. PI-negative and Annexin V-positive cells were considered early apoptotic cells. PI-positive and Annexin V-positive cells were considered dead cells.

### Cell proliferation assay

Cell Counting Kit-8 (CCK-8; Dojindo Molecular Technologies, Inc.) was used to assess cell proliferation. The cells were seeded in 96-well plates in triplicate at a density of ~ 1 × 10^3^ cells/well and were cultured for 1, 2, 3 and 4 days. After incubation, CCK-8 reagent was added for 2 h at 37 °C. Optical density (OD) was detected at 450 nm.

In the drug sensitivity test, cells were seeded in 96-well plates in triplicate at a density of ~ 1 × 10^3^ cells/well for 24 h at 37 °C, and separately treated with taxol (0, 5, 10, 20, 50, 80, 100, 200 μg/ml) and carboplatin (0, 10, 40, 80, 100, 150, 200, 500 μg/ml) for 48 h at 37 °C. Then, CCK-8 reagent was added for 2 h at 37 °C. Optical density (OD) was detected at 450 nm and the IC50 was calculated.

### Colony formation assay

The drug resistant cells (HO8910-TAX, HO8910-CBP, HO8910-TAX/CBP, SKOV3-TAX, SKOV3-CBP and SKOV3-TAX/CBP) and knockdown group cells (HO8910-TAX-shCON, HO8910-TAX-shGSTT1, HO8910-CBP-shCON, HO8910-CBP-shGSTT1, HO8910-TAX/CBP-shCON and HO8910-TAX/CBP-shGSTT1) were seeded in 6-well plates at a density of 1000 cells/well and incubated for 14 days. The cells were fixed with 4% paraformaldehyde at room temperature for 15 min and then stained with 0.5% crystal violet dye at room temperature for 10 min. The cell colony number was counted by microscope.

In the drug sensitivity test, the HO8910-TAX groups were treated with taxol (50 μg/ml), the HO8910-CBP groups were treated with carboplatin (100 μg/ml) and the HO8910-TAX/CBP groups were treated with taxol/carboplatin (50 μg/ml, 100 μg/ml) for 48 h at 37 °C. Then, the medium was replaced with complete culture medium and colony formation assays were performed.

### Immunohistochemistry

Chemotherapy sensitive and resistant SOC tissues were detected. Pathological section was immersed in Triton for 30 min. GSTT1 was detected as the primary antibody with a rabbit antibody at a dilution of 1:500 (Abcam, MA, USA). The ABC complex antibody was incubated at room temperature for 2 h. Immunohistochemical staining was carried out by chromogenic solution.

### Quantitative real-time PCR (qRT-PCR)

Total RNA was extracted from SKOV3 and HO8910 cells using Trizol reagent (Ambion, USA) according to the manufacturer’s instructions. cDNA was reverse transcribed from total RNA by a PrimeScript® RT reagent kit (TaKaRa, Japan) at 37 °C for 15 min and 85 °C for 5 s. The specific primers actin, GSTT1 and MDR1 used for qPCR were purchased from Shanghai Bio-engineering Ltd. (Table 1). PCR was performed with a SYBR Green PCR kit (Thermo Fisher Scientific, Rockford, USA) at 95 °C for 10 min, 95 °C for 15 s, 62 °C for 40 s, for 40 cycles. Gene-specific relative mRNA levels were calculated according to the standard eq. (2^-△△CT^ sample, 2^-△△CT^control).

### Western-blot and co-immunoprecipitation

For western blot, total protein was collected with RIPA buffer (Biyun Tian Biotechnology Co., Ltd.). Cell lysates were resolved by SDS-PAGE, and proteins were electro-transferred to polyvinylidene fluoride (PVDF) membranes (Millipore, USA). The PVDF membranes were blocked with 10% non-fat milk (Solarbio, Beijing, China). The primary antibodies included anti-GSTT1 (1:1000 dilution, Abcam, MA, USA), anti-BAX (1:1000 dilution, Abcam, MA, USA), anti-Bcl2 (1:1000 dilution, Abcam, MA, USA), anti-Cyclin B1 (1:1000 dilution, Abcam, MA, USA), anti-Cyclin E1 (1:1000 dilution, Abcam, MA, USA), anti-ERCC1 (1:1000 dilution, Abcam, MA, USA), anti-Topo I (1:1000 dilution, Abcam, MA, USA) and anti-MDR1 (1:2000 dilution, Arigo, MA, USA). The secondary antibody were sheep anti-rabbit and sheep anti-mouse (1:3000 dilution, Santa Cruz, Texas, USA). All proteins were stripped and re-probed with a β-actin antibody (1:3000 dilution, Abcam, MA, USA) as a loading control.

For co-immunoprecipitation, 1 mg of total protein was immunoprecipitated with diluted antibody in lysis buffer for 12 h. The antibody was eluted with 50 μL protein A and G beads. The beads were centrifuged for 5 min and the dried beads were mixed with 2X loading dye. The mixture was boiled for 10 min. The primary antibodies included anti-GSTT1 (1:40 dilution, Abcam, MA, USA) and anti-Topo I (1:40 dilution, Abcam, MA, USA). SDS-PAGE was then performed.

### Immunofluorescence

Cellular immunofluorescence was performed with anti-GSTT1 (1:400 dilution, Abcam, MA, USA) and anti-Topo I (1:500 dilution, Abcam, MA, USA) as primary monoclonal antibodies and sheep anti-rabbit and anti-mouse (1:3000 dilution, Thermo Company, USA) antibody as the fluorescence-labelled secondary antibody. DAPI was purchased from Shanghai RunJie Chemical Reagent Co., Ltd. Fluorescence densities were quantified by Image J software.

### Statistical analysis

Data were analyzed by GraphPad Prism statistical software. All data are shown as the means ± standard deviation (SD). The differences between multigroups were tested by ANOVA, and the differences between the two groups were tested by student’s t-tests. The *P* values smaller than 0.05 were considered statistically significant (* P < 0.05, * * P < 0.01, * * P < 0.001).

## Results

### Establishment of taxol / carboplatin-resistant (TAX/CBP) SOC cell lines

**Fig. 1:**
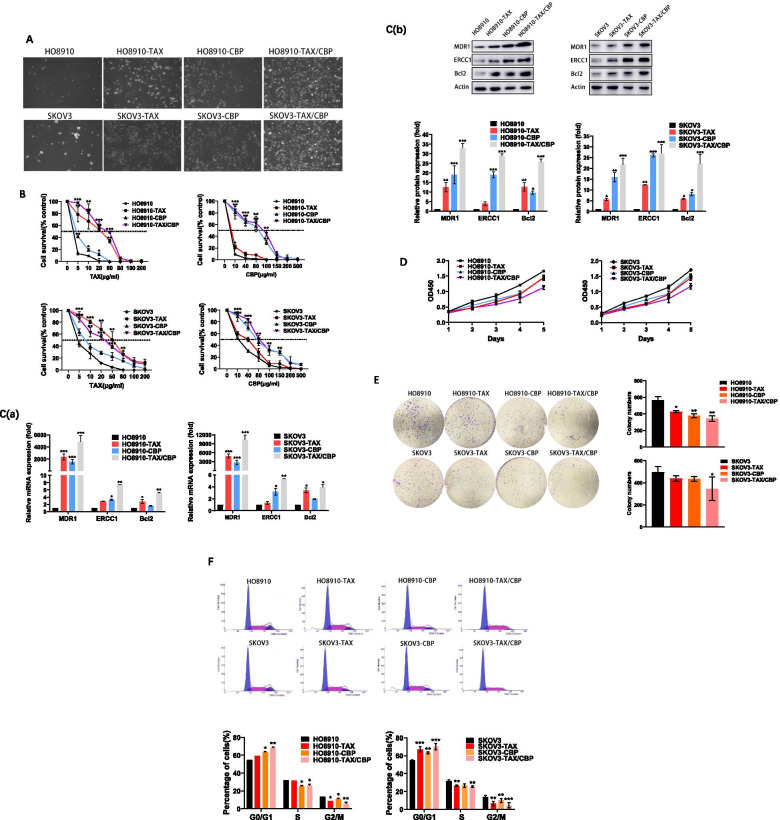
Establishment and characterization of taxol and carboplatin resistant SOC cell lines. **A** The growth of SOC cells (HO8910, HO8910-TAX, HO8910-CBP, HO8910-TAX/CBP, SKOV3, SKOV3-TAX, SKOV3-CBP and SKOV3-TAX/CBP) in the presence of treatment with 50 μg/ml taxol or (and) 100 μg/ml carboplatin for 5 days. **B** Cell survival rate was evaluated by CCK-8 assay in the presence of taxol or (and) carboplatin. Parental and taxol and carboplatin resistant cells were treated with different drug concentrations, then the 50% inhibitory concentration (IC50) was calculated. **C** qRT-PCR (a) and Western blot(b) analysis of the expression of MDR1, ERCC1 and Bcl2 in SOC cell lines. The mRNA and protein levels of analyzed genes were normalized by those of β-actin in the experiment. **D** The cell lines were grown in the absence of drug treatment, and the cell proliferation of SOC cells was tested by CCK-8 assay. **E** The cell lines were grown in the absence of drug treatment, and the colony formation of SOC cells was assessed. **F** Cell cycle analysis by flow cytometry on TAX or (and) CBP-resistant and parental cells, when they are grown in the absence of drug treatment. *P < 0.05, **P < 0.01, ***P < 0.001. Data in this figure are presented as the mean ± SD of three independent experiments

To explore the mechanism of combined resistance of taxol and carboplatin in SOC cell lines, taxol / carboplatin-resistant serous ovarian cancer (SOC) cells, SKOV3-TAX/CBP and HO8910-TAX/CBP were screened in the presence of long-term drug treatment, two different parental cell lines, SKOV3 and HO8910 being used in the experiments. Morphologically, the taxol / carboplatin-resistant SOC cell lines were identical to their parental cell lines. However, the parental cells, SKOV3 and HO8910 were mostly killed in the presence of 50 μg/ml taxol and 100 μg/ml carboplatin for 5 days, while taxol-resistant cells, SKOV3-TAX and HO8910-TAX, and carboplatin-resistant cells, SKOV3-CBP and HO8910-CBP, were resistant to the treatments to some extent. As expected, double resistant cell lines, SKOV3-TAX/CBP and HO8910-TAX/CBP showed much more viability, compared to single resistant cells and parental cells (Fig. 1A).

Furthermore, the survival rates of the SOC cells were examined over 2 weeks with different drug concentrations. Two double resistant cell lines, HO8910-TAX/CBP and SKOV3-TAX/CBP showed significantly higher survival rates than those of control cells, after treated with different drug concentrations of taxol and carboplatin. When treated with taxol, the IC50s for HO8910, HO8910-TAX, HO8910-CBP, HO8910-TAX/CBP cells were 3.1 ± 1.53, 31.2 ± 3.54, 5.1 ± 0.37, 45.6 ± 2.14 μg/ml, respectively, and the IC50s for SKOV3, SKOV3-TAX, SKOV3-CBP and SKOV3-TAX/CBP were 3.5 ± 1.15, 50.2 ± 2.28, 7.1 ± 1.33 and 35.1 ± 3.31 μg/ml, respectively. While treated with carboplatin, the IC50s for HO8910, HO8910-TAX, HO8910-CBP, HO8910-TAX/CBP cells were 8.1 ± 0.34, 9.2 ± 2.55, 85.3 ± 2.53, 93.6 ± 5.28 μg/ml, respectively, and the IC50s for SKOV3, SKOV3-TAX, SKOV3-CBP and SKOV3-TAX/CBP were 9.9 ± 1.21, 39.2 ± 2.11, 75.9 ± 5.47 and 81.6 ± 3.24 μg/ml, respectively (Fig. 1B).

Since the TAX/CBP resistant SOC cell lines were functionally established, then some inducible drug resistance-related molecules were detected by qRT-PCR and western blot. As previously showed, MDR1, ERCC1 and Bcl2 were induced by multiple drug treatment, including taxol or carboplatin [11–14]. As Fig. 1C shown, both mRNA and protein levels of MDR1, ERCC1 and Bcl2 expression were clearly increased in TAX/CBP-resistant cells, and double resistant cells showed higher expression than single resistant cells and parental cells. This data indicated these cell lines were molecularly TAX/CBP resistant cell lines.

Subsequently, the biological phenotypes of TAX/CBP-resistant cells were further examined. As Fig. 1D and E shown, SOC cell lines were grown in the absence of taxol or carboplatin. Compared with the parent cells HO8910 and SKOV3, the proliferation of resistant cells was modestly compromised, while clone formation abilities of TAX/CBP-resistant cells were obviously inhibited, which indicating less viability for TAX/CBP resistant cell lines under normal condition. Moreover, cell cycle analysis by flow cytometry demonstrated that more taxol and carboplatin resistant SOC cells were arrested at the G0/G1-phase, and less taxol and carboplatin resistant cells were committed to S phase and G2/M phase, particularly TAX/CBP-resistant cells (*P* < 0.001) (Fig. 1F). These results concluded that the TAX/CBP resistant cell lines gained the phenotype of more quiescence status with inhibited cell proliferation in the absence of treatment.

### GSTT1 is substantially upregulated in taxol / carboplatin-resistant SOC cells

**Fig. 2:**
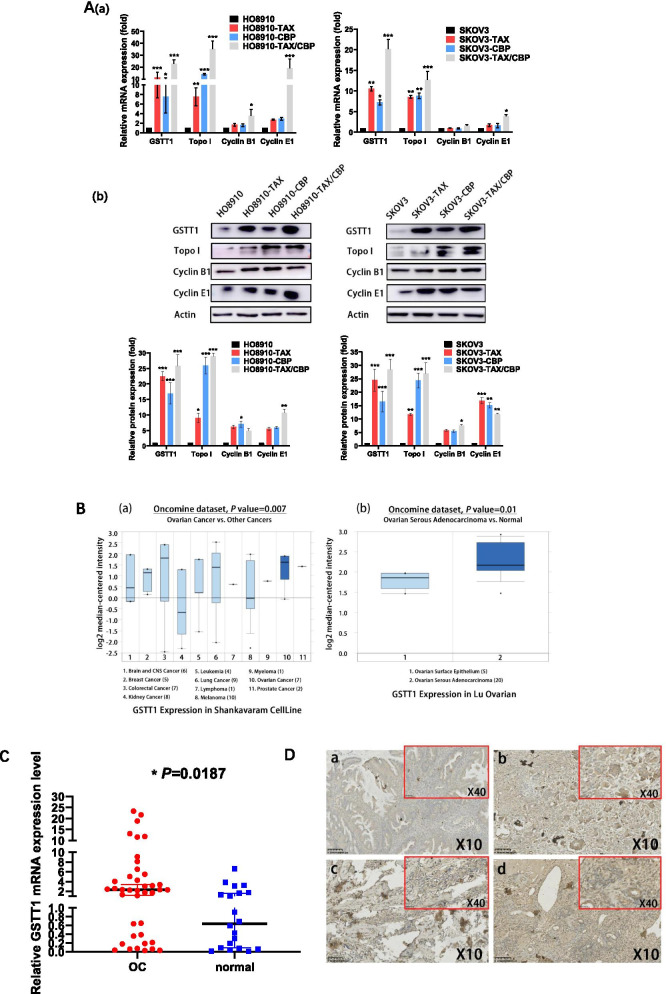
Overexpression of GSTT1 in drug-resistance SOC. A. The expression level of GSTT1 and other taxol and carboplatin resistance-related molecules was assessed by qRT-PCR(a) and Western blot(b). GSTT1, Topo I, and Cyclin E1 were up-regulated in drug-resistant HO8910 and SKOV3 cells. B. Oncomine data analysis for GSTT1 in SOC. (a) GSTT1 was over-expressed in SOC, compared to the most of other cancer samples. (b) GSTT1 was over-expressed in SOC, compared to normal tissues. C. The transcriptional level of GSTT1 in OC tissues (*n* = 40) and normal tissues (*n* = 20) were determined by qRT-PCR and normalized by the internal control β-actin gene. The bars in the figure indicated the median of the relative expression of GSTT1. D. Immunostaining of GSTT1 protein with GSTT1 antibody in SOC tissues (a-b): Case 1, TAX-sensitive SOC showed weak staining of GSTT1, while TAX-resistant SOC showed much more positive staining of GSTT1. (c-d): Case 2, TAX-sensitive SOC(c) and -resistance SOC (d) showed images of weak and strong GSTT1 expression, respectively

Since taxol / carboplatin resistant cell lines have been developed, the expression level of drug-resistant genes was investigated. Previous studies indicated DNA damage repair genes Topo I [15, 16] and cell cycle genes such as Cyclin B1 and Cyclin E1, are related to chemoresistance [17–19]. It was also shown that Glutathione-S-transferases-theta1 (GSTT1), could be cancer related gene. To find the situation in the scenario of TAX/CBP treatment, we tested those expression in TAX/CBP chemoresistant cells. As shown in Fig. 2A, the mRNA and protein expression of Topo I and Cyclin E1 was significantly up-regulated in taxol / carboplatin resistant cell lines, but that of Cyclin B1 was just slightly up-regulated. Specially, the expression level of GSTT1 was substantially increased in all drug-resistant cell lines, compared to other genes. Furthermore, we performed Oncomine database (http://www.oncomine.org/) searching to analyse the expression of GSTT1 in different cancer samples. Interestingly, GSTT1 was particularly overexpressed in ovarian and colorectal cancer, and to a lesser extent expressed in breast, lung and prostate cancer and myeloma, compared to other cancers (*P* = 0.007) (Fig. 2B). As expected, GSTT1 expression in ovarian normal tissues is lower than that in cancer cells. In total, it indicated that not only was this gene a cancer related gene that could be involved in cancer development or progression, but also it could be a chemoresistant gene in SOC.

To investigate the physiological expression level of GSTT1, 40 SOC patient tissues and 20 normal ovarian tissues were analyzed. As shown in Fig. 2C, GSTT1 mRNA levels were significantly higher in SOC patient tissues than those in normal tissues (*P* = 0.0187). For further verification, we picked up the SOC tissues for IHC staining by GSTT1 specific antibody. These SOC tissues were from two different SOC patients who underwent operations twice, before and after chemotherapy with taxol, respectively (Fig. 2D). In case 1, the expression of GSTT1 was marginal in the tissue before chemotherapeutical treatment. In the contrast, the expression was positive strongly after chemotherapeutical treatment. In case 2, the expression of GSTT1 was also positive in the tissue before treatment. However, after chemotherapeutical treatment, the expression was significantly stronger than before treatment. Conclusively, GSTT1 expression was physiologically up-regulated in taxol / carboplatin-resistant ovarian cancer cells.

### Down-regulation of GSTT1 increases the sensitivity of drug-resisitant cells to taxol / carboplatin

**Fig. 3:**
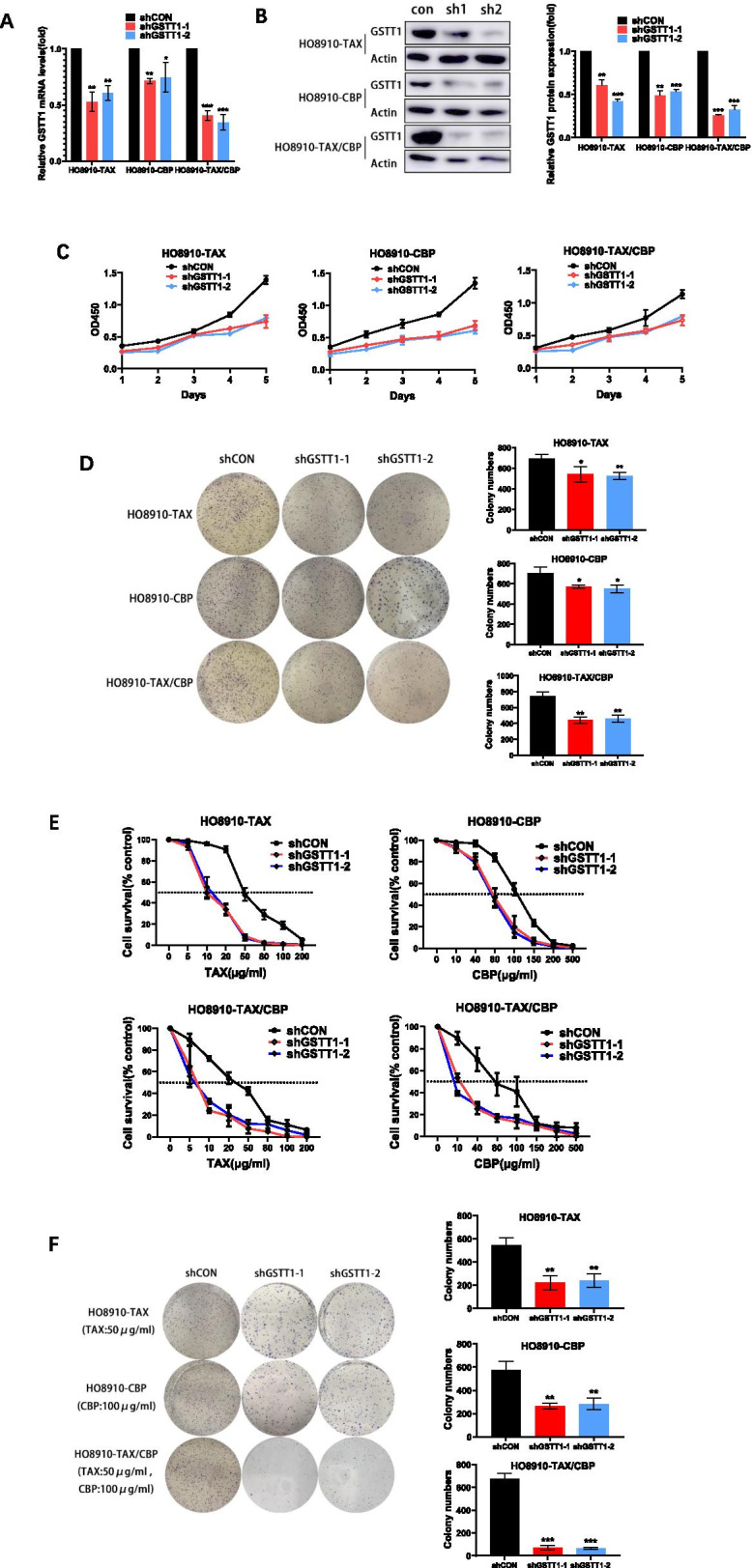
Down-regulation of GSTT1 increased the sensitivity of drug-resistant cells to taxol and carboplatin. Knockdown efficiency of GSTT1 was determined by qRT-PCR analysis (**A**) and Western-blot (**B**) in HO8910 cells resistant to taxol or (and) carboplatin. **C** The proliferation of HO8910 stable cell lines was tested by CCK-8 assay in the absence of taxol and carboplatin. **D** The colony formation was assessed using HO8910 stable cell lines in the absence of taxol and carboplatin. **E** Inhibition of GSTT1 increased the sensitivity of taxol and carboplatin resistant cell lines to the treatment of 50 μg/ml taxol or 100 μg/ml carboplatin, or to combined treatment of these two drugs for 48 h, in CCK-8 assays and colony formation assays (**F**)

To explore the potential functions of GSTT1 in SOC drug-resistant cells, lentivirus-mediated RNAi was used to down-regulate the expression of GSTT1. As Fig. 3 A and B show, both the mRNA and protein expression of GSTT1 were significantly inhibited in the taxol / carboplatin-resistant cells. The data indicated that the knockdown efficiency of the RNAi was satisfactory. The stable transfected cells were selected for subsequent experiments.

Then, the effect of GSTT1 on the proliferation of drug-resistant cells was investigated. Compared with control cells (shCON), the proliferation of GSTT1 knockdown cells was obviously inhibited in HO8910 drug-resistant cells without drug treatment (Fig. 3C). Furthermore, this notion was confirmed by cell colony formation assays. As shown in Fig. 3D, the colony numbers of GSTT1 knockdown cells were less than that of control cells under the condition of no treatment (*P* < 0.05), especially the knockdown in double resistant cells. The results indicated that down-regulation of GSTT1 depressed the viability and proliferation of TAX/CBP-resistant cells in the absence of taxol and carboplatin treatment.

Finally, to clarify the impact of GSTT1 on drug sensitivity, taxol and carboplatin-resistant SOC cells were treated with these two drugs respectively. As treated with taxol in the experiment of Fig. 3E, the IC50 values of HO8910-TAX cells were clearly reduced from 48.1 ± 9.39 in control cells to 9.2 ± 4.68 and 15.2 ± 2.21 μg/ml in GSTT1 knockdown cells, and the IC50 values of HO8910-TAX/CBP cells was also decreased from 35.2 ± 3.37 to 8.2 ± 1.61 and 7.3 ± 1.22 μg/ml. As treated with carboplatin, the IC50 values of HO8910-CBP cells were decreased from 118.2 ± 31.33 to 80.1 ± 3.52 and 75.2 ± 3.11 μg/ml, and from 79.1 ± 6.83 to 18.6 ± 1.72 and 9.4 ± 1.16 μg/ml in HO8910-TAX/CBP cells (Fig. 3E). Further, colony formation assays were also performed. The HO8910-TAX groups were treated with taxol (50 μg/ml), HO8910-CBP with carboplatin (100 μg/ml) and HO8910-TAX/CBP with taxol and carboplatin (50 μg/ml and 100 μg/ml, respectively) for 48 h. Compared with the control, the knockdown of GSTT1 clearly reduced the colony formation in all three lines of HO8910-TAX, HO8910-CBP and HO8910-TAX/CBP cells respectively when treated with taxol, carboplatin and taxol / carboplatin (Fig. 3F). Overall, the knockdown of GSTT1 gene obviously lead to decreased cell survival and proliferation under the treatment of TAX and CBP.

### Down-regulation of GSTT1 increases cell death and induces cell cycle arrest

**Fig. 4:**
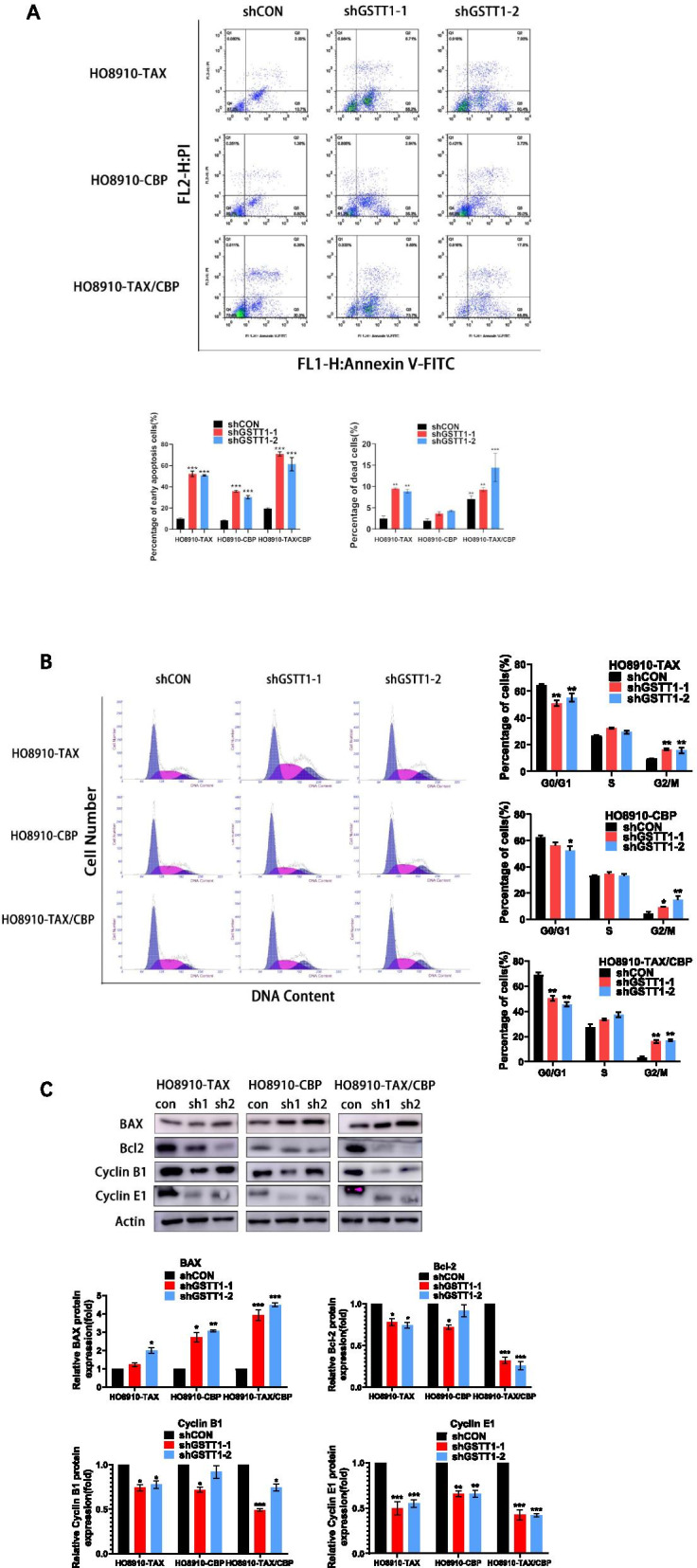
Down-regulation of GSTT1 increased apoptosis and induced cell cycle arrest. **A** Inhibition of GSTT1 facilitated apoptosis in untreated taxol and carboplatin resistant HO8910 cells. **B** Knockdown of GSTT1 in drug-resistant HO8910 cells induced cell cycle arrest at G2/M-phase. **C** Molecules related to apoptosis and cell cycle were analyzed by Western blot

Since GSTT1 had an impact on viability and proliferation of SOC cells, we wondered if it could mechanistically play roles in cell death and cell cycle in these cells. Thus, Annexin V staining and flow cytometric analysis were performed to monitor cell death in GSTT1 knockdown cells resistant to taxol and carbopaltin under the condition of no treatment. As illustrated in Fig. 4A, down-regulation of GSTT1 in drug-resistant cells resulted in a high percentage of early apoptotic cells, as compared to the control cells HO8910-TAX, HO8910-CBP and HO8910-TAX/CBP. To a lesser extent, knockdown of GSTT1 also increased cell death in the knockdown cells than control cells, especially in double resistant cells.

Then, the cell cycle analysis of drug-resistant cells without treatment was measured by flow cytometry. The results showed that suppression of GSTT1 promoted cell cycle arrest at G2/M-phase in double-resistant cells, as well as in single resistant cells to a lesser extent (Fig. 4B). Correspondingly, the expression levels of cell cycle-associated protein Cyclin E1 were significantly down-regulated in GSTT1-silenced HO8910 cells, but those of Cyclin B1 were just down-regulated to a lesser extent. Interestingly, it appeared that the levels of pro-apoptotic protein BAX were marginally increased, while those of anti-apoptotic Bcl2 were substantially decreased [14, 20], indicating that GSTT1 involved in not only cell cycle pathway, but also apoptotic pathway (Fig. 4C).

### The role of GSTT1 in drug-resistance is related to DNA damage repair molecules

**Fig. 5:**
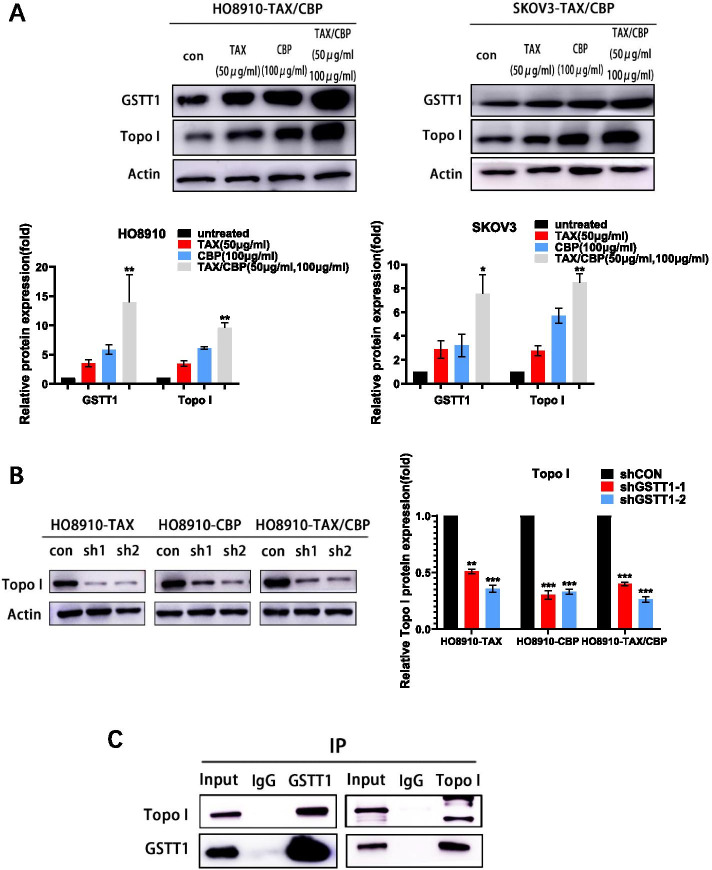
Down-regulation of GSTT1 decreased DNA damage repair molecules Topo I. **A** Western blot was used to detect the expression of GSTT1 and Topo I protein in taxol / carboplatin-resistant ovarian cell lines treated with taxol and carboplatin for 48 h. **B** Topo I protein levels in shGSTT1–1/2 transfected cells were analyzed. C. GSTT1 interacted with Topo I in co-immunoprecipitation experiment

The alternation of molecules related to metabolism and DNA damage repair were pathological features of drug resistance in cancer cells, and some metabolic enzyme such as GSTs could defend cells through enhancing DNA repair. To test if and how GSTT1 is related to DNA repair machinery such as DNA topoisomerase, in drug resistant SOC cells, the taxol / carboplatin double resistant cell lines HO8910-TAX/CBP and SKOV3-TAX/CBP were treated with taxol (50 μg/ml) and carboplatin (100 μg/ml) for 48 h. The results showed that taxol and carboplatin could induce the up-regulation of GSTT1 and DNA topoisomerase 1 (Topo I) synergistically, in the presence of single or combined treatment of taxol / carboplatin (Fig. 5A). Interestingly, suppression of GSTT1 resulted in decrease expression of Topo I significantly (Fig. 5B). To further clarify the relationship of these two molecules, the co-immunoprecipitation experiment was performed. The result revealed that GSTT1 interacted with Topo I in vitro (Fig. 5C). The data indicated that GSTT1 was related to DNA damage repair molecule Topo I, and could involve in DNA damage repair process in SOC drug resistant cells.

## Discussion

The 5-year survival rate of ovarian cancer patients is less than 50%. Ovarian cancer is also known as the “top killer” of gynecological malignancies, which seriously threatens women’s health. After standardized treatment, ovarian cancer often recurs and metastasizes within 3–5 years. Glutathione S-transferases (GST) is a family of phase II metabolic enzymes with the ability to catalyse the conjugation of reduced form of glutathione (GSH) to a variety of toxic compounds including chemotherapeutic agents [21]. Therefore, the interaction between GSTs and their substrates such as chemotherapeutic drugs for cancer treatment could affect the metabolism of these drugs, through which affecting the death and chemoresistance of cancer cells. In fact, recent studies indeed showed GSTs played roles in cancer development, progression and drug resistance [22]. Some studies showed that the polymorphisms of a major GST family member GSTT1, were associated with some types of cancer, such as acute lymphoblastic leukemia, prostate cancer, or associated with higher tumor grade in colorectal cancer [23–26]. But other research argued that GSTT1 were not associated epithelial ovarian cancer, or the survival or clinical response in colorectal cancer and esophageal cancer [27–29]. Altogether, how GSTT1 plays its roles in cancer development and chemoresistance is quite controversial, and its specific roles in serous ovarian cancer remain unknown before our study.

In this study, we showed here GSTT1 was significantly upregulated in both established chemoresistant SOC cell lines and patient tissues after chemotherapeutical treatment, indicating its biological functions relevance to SOC. Up-regulation of GSTT1 was associated with SOC cell survival after chemotherapeutical treatment, and suppression of GSTT1 expression would inhibit the proliferation of SOC cells and rescue the sensitivity to taxol / carboplatin. Meanwhile, down-regulation of GSTT1 accelerated the number of resistance cell apoptosis after treatment with taxol / carboplatin, and the typical quiescence status of cell cycle arrest was transformed into division status. As expected, down-regulated of GSTT1 expression induced the expression changes of apoptosis pathway related proteins with increase of BAX and decrease of Bcl2. Additionally, the expression levels of Cyclin B1 and Cyclin E1 were both declined significantly after down-regulation of GSTT1 in resistance cells. It was documented that GST isozymes interacted with the members of mitogen-activated protein kinase (MAPK) pathways involved in cell-survival and cell death signalling mechanisms [14, 30]. Through regulating cell cycle and cell death, GSTT1 suppression causes more vulnerability of SOC cells to chemotherapeutics, suggesting its role in enhancing SOC chemoresistance. Therefore, it can be reasonably expected that dysfunction of GSTT1 in vivo could increase sensitivity of SOC patients to chemotherapy in the future studies.

DNA topoisomerases are nuclear enzymes which modify DNA topology and have critical function in DNA replication and DNA repair [31, 32]. Many cancer chemotherapeutics target and inactivate DNA topoisomerases so as to intervene the replication of cancer cells, through which causing cell death after treatment [31]. To explore the molecular mechanism of GSTT1’s role in chemoresistance, it is reasonable to ask if DNA topoisomerases are potential targets of GSTT1. It was also reported that DNA topoisomerase family member Topo I activity was inhibited via induction of the MAPK signal transduction pathway. In addition, Topo I induced the phosphorylation of phospholipase Cγ1, c-Raf, ERK-1/2, and p38 MAPK, that stimulated fibroblast migration via a G protein-coupled receptor [33, 34]. As far as the molecular mechanism of GSTT1 is concerned, the synergistic expression between GSTT1 and Topo I in SOC resistance cells suggests that there may be potential interaction between two proteins. Our data showed that GSTT1 suppression resulted in the down-regulated of Topo I, further co-immunoprecipitation experiments revealed that the interaction between GSTT1 and Topo I was existed in vitro distinctly. Thus, the results suggest that GSTT1 may involve in DNA repair during chemotherapeutical treatment with a non-enzymatic function in SOC cells. So far, how and why this interaction happens in vivo is still not known. However, there is the possibility that the cofactors in the Topo I complex could be the substrates of GSTT1. During the progression of cell cycle, the interplay between GSTT1 and its substrate in DNA repair machinery results in more quiescence cells and thus blocks cell division, through which GSTT1 could provide cell defence to drug treatment and play roles in chemoresistance.

## Conclusion

In conclusion, GSTT1 could be a chemoresistance related gene in serous ovarian cancer cells. It’s physiologically upregulated during chemotherapeutical treatment, which leads to more cell survival and less cell division in SOC cells. Suppression of its expression promotes cell division and increases the sensitivity to drug treatment. In summary, GSTT1 is a potential target for serous ovarian cancer treatment to overcome chemoresistance in order to improve the survival rate.

## Data Availability

The datasets generated during and/or analysed during the current study are available from the corresponding author on reasonable request.
